# Multi-periodic photonic mechanism for non-spectral magenta coloration in Anna's hummingbird (*Calypte anna*) feathers

**DOI:** 10.1098/rsif.2026.0601

**Published:** 2026-10-28

**Authors:** Sangyeop Kim, Jihun Kang, Seunghwan Moon, Liliana D'Alba, Matthew D. Shawkey, Jong-Souk Yeo

**Affiliations:** ^1^ School of Integrated Technology, College of Computing, Yonsei University, Incheon, Republic of Korea; ^2^ BK21 Graduate Program in Intelligent Semiconductor Technology, Yonsei University, Seoul, Republic of Korea; ^3^ Evolution and Optics of Nanostructures Group, Department of Biology, Ghent University, Ghent, Belgium; ^4^ Naturalis Biodiversity Centre, Leiden, The Netherlands

**Keywords:** structural colours, non-spectral, multi-periodic, melanosomes, hummingbirds

## Abstract

Hummingbird feathers generate iridescent colours through light interference within multi-layered arrays of platelet-shaped melanosomes embedded in corneous beta-protein. In Anna's hummingbird (*Calypte anna*), the gorget feathers produce a non-spectral magenta hue with multiple reflectance peaks in the red and blue spectral regions. The primary red peak has been attributed to the melanosome stack; however, the optical mechanism underlying the secondary blue peak remains unexplained. Here, we identify a previously uncharacterized intermediate-sized melanosome layer and demonstrate that it functions as part of a multi-periodic multi-layer reflector, establishing its essential role in secondary blue peak formation. Electron microscopy and tomography revealed a continuous melanosome size gradient across the barbule depth, with a superficial small melanosome, two intermediate-sized melanosomes and progressively larger melanosomes in deeper layers. Optical simulations indicate that the secondary blue peak depends on optical interaction between the top and middle layers, while the deeper multi-layer strengthens and sharpens the dominant red peak through multi-layer interference. Oxygen plasma etching showed that removal of the superficial layer eliminated the blue peak, further supporting this mechanism. Our findings reveal that the non-spectral magenta coloration arises from hierarchical optical interactions across graded melanosome architecture, providing a biomimetic design principle for non-spectral reflectors.

## 1. Introduction

The vivid plumage colours of birds are produced by pigments, nanostructures or their combination, a diversity of mechanisms that have made avian coloration a model system for studying the physical basis of colour production in nature [1–5]. Pigmentary colours originate from molecules that absorb specific wavelengths of light, whereas structural colours result from wavelength-selective scattering or interference by nanoscale architecture. Structural colours are classified as iridescent and non-iridescent. Iridescent colours, which change with illumination or viewing angles, are typically produced by periodic nano structures, such as thin films, diffraction gratings and photonic crystals [6–10]. By contrast, non-iridescent colours are angle-independent and generally result from coherent scattering by quasi-ordered nano- structures [11–14].

Pennaceous feathers consist of a central rachis bearing paired barbs, each with smaller branches called barbules [15,16]. Iridescent structural coloration in feather barbules typically arises from photonic crystal-like arrangements of melanosomes—organelles that synthesize and store melanin—embedded within feather corneous beta-protein (C*β*P; formerly beta-keratin) and/or air [17–20]. Melanosomes occur in various morphologies, including spherical, rod-shaped, hollow rod, platelet and hollow platelet forms [21,22]. Among these, hollow melanosomes can enhance colour brightness and saturation because air-filled cavities increase the refractive index contrast between air (*n* = 1.0) and C*β*P (*n* = 1.56), thereby increasing scattering [23–25].

Hummingbirds are especially well known for their vivid iridescent plumage, which is generated by multi-layer arrangements of mostly porous, platelet-shaped melanosomes embedded in C*β*P in their feather barbules [26–29]. Previous studies have characterized two melanosome size classes in hummingbird barbules: smaller melanosomes forming the outermost layer and larger melanosomes composing the underlying stacks [30,31]. In Anna's hummingbird (*Calypte anna*), the iridescent magenta crown and gorget feathers exhibit multiple reflectance peaks, including a dominant red and secondary blue peak [30,32]. This hue is classified as a non-spectral colour, as it cannot be defined by any single point on the spectrum but instead arises from the simultaneous presence of multiple reflectance peaks [33,34]. Hummingbirds can discriminate such non-spectral colours, which play an essential role in visual signalling and foraging behaviour [35]. The secondary blue peak has been attributed to the optical interaction between the C*β*P cortex and the superficial small melanosomes, a relationship confirmed across multiple hummingbird species using optical models [31]. However, in Anna's hummingbird, optical models that treat the barbule melanosome stack as consisting of only two size classes (small superficial melanosomes and uniform underlying melanosomes) failed to reproduce a discrete secondary blue peak [30]. This discrepancy points to an additional, previously unresolved structural component that governs the non-spectral magenta coloration. Here, we investigate the photonic nanostructures of Anna's hummingbird using electron microscopy and tomography, optical simulations with one-dimensional and three-dimensional modelling and plasma etching, revealing a previously uncharacterized intermediate melanosome layer distinct in size from both the superficial small melanosomes and the deeper large melanosomes. The optical interaction between this intermediate layer and the top layer is shown to be necessary and sufficient to generate the secondary blue reflectance peak underlying the non-spectral magenta coloration.

## 2. Methods

### 2.1. Feather samples

Gorget feathers from Anna's hummingbird were provided by Melissa Meadows from a captive population [36].

### 2.2. Optical measurement and characterization

Optical microscopic images were captured using an optical microscope (Eclipse Ti-U, Nikon). Reflectance spectra of the barbules were measured over an area of 6.2 × 6.2 µm using a microspectrophotometer (20/30PV, Craic Technologies). The spectra were recorded in the UV to visible wavelength range (300–800 nm) using a 40× objective lens (numerical aperture (NA) = 0.6) under normal incidence illumination from a Xe lamp. An aluminium mirror served as the white reference for all spectroscopic measurements. Commission Internationale de l'Éclairage (CIE) chromaticity coordinates were calculated from the measured reflectance data using the CIE 1931 standard observer and D65 standard illuminant in Origin Pro (OriginLab, USA) to characterize the reflectance spectra.

### 2.3. Electron microscopy

Scanning electron microscopy (SEM) images of Anna's hummingbird barbules were acquired at 10 kV using a field emission scanning electron microscope (JSM-7100F). Cross-sectional SEM images were acquired using the backscattered electron detector (BSD) of a scanning electron microscope (Crossbeam 550). A sample for cross-sectional SEM imaging was prepared using a resin embedding procedure. Anna's hummingbird barbules were fixed overnight at 4°C using Karnovsky's fixative. After fixation, the samples were rinsed three times for 5 minutes each with 0.05 M sodium cacodylate buffer at room temperature. Barbules were post-fixed with 1% osmium tetroxide for 1 hour at 4°C. The samples were then washed three times with distilled water. For en bloc staining, the barbules were incubated in 0.5% uranyl acetate overnight at 4°C, and then washed again with distilled water. The samples were dehydrated through a graded ethanol series (30–100%), treated with propylene oxide for 15 minutes, followed by two additional 15-minute treatments with pure propylene oxide. For embedding, a 3-hour transition mixture of propylene oxide and Spurr's resin (Electron Microscopy Sciences, USA) was applied. Subsequently, the samples were infiltrated with resin and embedded overnight at 70°C. Ultrathin sections (100 nm thick) were prepared using an ultramicrotome equipped with a diamond knife.

### 2.4. Structural characterization

We measured the average thickness of the nanostructural components in the barbules from micrographs using ImageJ software (supplementary material, figure S1). The measured components included: (a) melanosomes, (b) air vesicles, (c) melanosome membranes, (d) C*β*P, and (e) the C*β*P cortex. Since melanosomes are flat with numerous internal air cavities, we measured the thickness of melanosomes (a) and air vesicles (b) from multiple sections of a single melanosome and calculated the average thickness. (c) Melanosome membrane thickness was calculated by subtracting (b) the air vesicle thickness from (a) the melanosome thickness and dividing by 2. An average of 14 melanosomes per layer (10–19) was measured, totalling 199 melanosomes. For (d) the C*β*P layer and (e) cortex, thickness was measured between or above individual melanosomes. An average of 29 layers per region (19–30) was measured, totalling 403 layers. Average thicknesses for the three components in each layer were: top (cortex (e = d_1_): 26 nm, melanosome (a_1_): 108 nm, air vesicle (b_1_): 42 nm), middle (C*β*P (d_2_): 23 nm, melanosome (a_2_): 189 nm, air vesicle (b_2_): 119 nm) and bottom layers (C*β*P (d_3_): 27 nm, melanosome (a_3_): 222 nm, air vesicle (b_3_): 152 nm). Table S1 in the supplementary material presents the average thickness for each of the 14 layers.

### 2.5. Three-dimensional tomographic imaging

Three-dimensional tomographic imaging proceeded through four stages: sample preparation, ultrathin sectioning, imaging and reconstruction. For sample preparation, the samples were processed based on the same resin-embedding method applied for cross-sectional SEM imaging. For ultrathin sectioning, the resin-embedded barbules were sectioned into 100 nm ultrathin slices using an ultramicrotome and mounted on silicon wafers. Imaging was conducted using a field emission scanning electron microscope (AURIGA, Carl Zeiss) at 5 kV. Images of the nanostructures were captured, and regions of interest were identified using Atlas 5 software (Carl Zeiss). Finally, the image series was reconstructed into a three-dimensional tomographic model using commercial software (Amira-Avizo, Thermo Fisher). Our three-dimensional model was reconstructed using a binary segmentation technique that classifies voxels as either void (internal air vesicle) or solid material. Since melanin and C*β*P could not be distinguished at the available contrast levels, they were treated as a single unified material imported into the optical simulation.

### 2.6. Finite-difference time-domain simulations

Finite-difference time-domain (FDTD) simulations were performed using commercial software (Ansys-Lumerical FDTD, Ansys, USA) to compare the reflectance spectra. In the one-dimensional model-based simulation, wavelength-dependent complex refractive indices of melanin and C*β*P were adopted from published parameter values and modelled using the following expression:

(2.1) $$\;\tilde{n}=\;A+\frac{B}{\lambda^{2}}-iCexp\left(-\frac{\lambda }{\lambda_{i}}\right),$$

where *λ* indicates the light wavelength [31]. Parameter values for melanin were adopted from previous studies (*A* = 1.648, *B* = 23 700, *C* = 0.56 and *λ_i_* = 270 nm), and for C*β*P, *A* = 1.532 and *B* = 5890 [37,38].

The internal air vesicle layers, composed of melanin, air and C*β*P, were assigned effective refractive indices calculated based on the volume fraction values reported in a previous study [30]. The volume fractions of melanin (*f_m_*), air (*f_a_*) and C*β*P (*f_k_*) were 0.4, 0.55 and 0.05, respectively. The effective refractive index (*n*_eff_) was calculated using the following formula:

(2.2) $$\;n_{eff}=f_{k}\;n_{k}+f_{m}(n_{m}-ik_{m})+f_{a}n_{a},$$

where *n_k_*​, *n_m_* and *n_a_*​ indicate the refractive indices of C*β*P, melanin and air, respectively, and *k_m_* is the extinction coefficient of melanin.

In models 1 and 3, the refractive index of each constituent material was used, whereas in models 2 and 4, a complex refractive index was employed, calculated from the thickness ratio of the melanosome membrane (73%) and C*β*P (27%).

In the three-dimensional tomographic imaging-based FDTD simulation, periodic boundary conditions (2 × 2 µm^2^) were applied along the x- and y-axes, and perfectly matched layers (PMLs) boundary conditions (7 µm) along the z-axis, to capture all reflected light. Structures were modelled using the same complex refractive index as in models 2 and 4.

### 2.7. Etching of the feather

Feathers were mounted flat on a glass substrate using carbon tape, with the dorsal side oriented upward to ensure uniform exposure during etching. Oxygen plasma etching was then applied to the feather barbules by introducing oxygen gas into the plasma chamber (ASS-5000, SNTek). The process was conducted at a constant power (50 W), pressure (400 mTorr) and oxygen gas flow rate (40 standard cm^3^ min^−1^) [39]. The etched layer was controlled by varying etching time (60 and 120 s), starting from the original (untreated) feather barbules.

## 3. Results and discussion

### 3.1. Optical appearance and structural origin of coloration in Anna's hummingbird

The crown and gorget feathers of the male Anna's hummingbird (*C. anna*) have vivid magenta coloration (figure 1a). Reflectance spectra of individual gorget barbules as measured by a microspectrophotometer (figure 1b,c) revealed a multi-peak reflectance producing a non-spectral colour. The dominant reflectance peak appeared at approximately 632 nm, with a maximum reflectance of approximately 32.8%. A secondary peak occurred near 460 nm, with a reflectance of approximately 12.0%, corresponding to the red and blue spectral regions, respectively. Because the red peak is roughly 2.7 times higher, the resulting hue appeared red-shifted toward magenta. For clarity and consistency, this colour is hereafter referred to simply as magenta.

**Figure 1.**
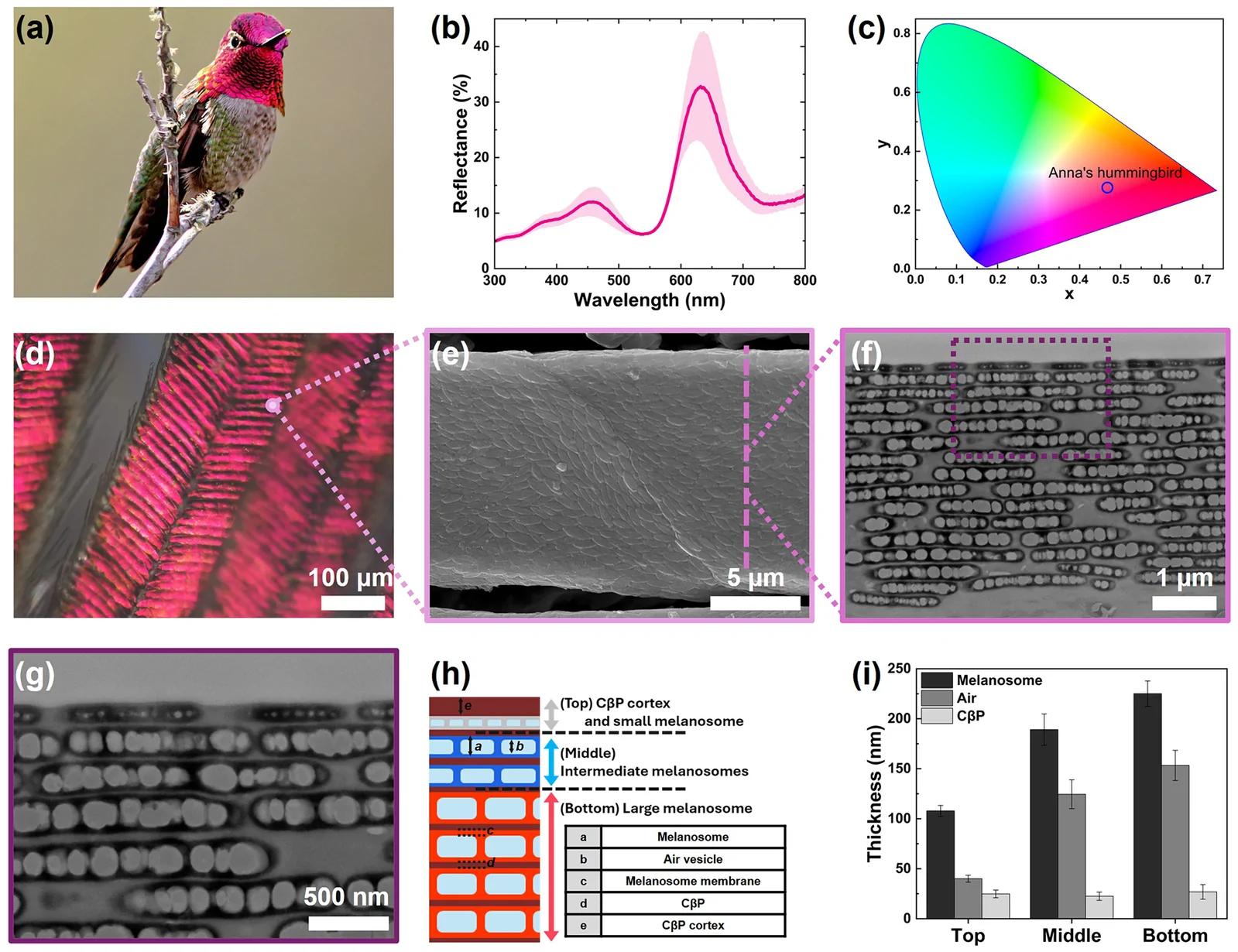
Bright magenta coloration and nanostructure of Anna's hummingbird. (a) Photograph of Anna's hummingbird showing magenta crown and gorget feathers. Photo credit: Alan Schmierer/USFWS (public domain, fws.gov). (b) Reflectance spectrum of gorget barbules measured using a microspectrophotometer; shaded areas indicate s.d. (c) CIE chromaticity diagram of Anna's hummingbird gorget feathers. (d) OM image of magenta barbules in the gorget feather. (e) SEM image of the surface of a barbule. (f) Cross-sectional SEM image revealing nanostructures of a barbule. (g) Magnified SEM image of the region indicated with the dotted box in (f). (h) Schematic illustration and table of structural parameters, including melanosome, air, melanosome membrane, C*β*P and C*β*P cortex. (i) Measured thicknesses of the melanosome, air and C*β*P layers (melanosome and air, *N* = 199; C*β*P, *N* = 403); error bars represent the s.d. Abbreviations: CIE, Commission Internationale de l'Éclairage; OM, optical microscopy; SEM, scanning electron microscopy; C*β*P, corneous beta-protein; N, number of measurements.

Under optical microscopy (OM), the dorsal surface of each barbule appeared magenta (figure 1d). The top view of the SEM revealed a densely packed, disordered arrangement of platelet-shaped melanosomes on the surface of the barbule (figure 1e). Cross-sectional BSD (SEM-BSD) imaging revealed these small surface melanosomes together with 12–14 underlying layers of larger melanosomes (figure 1f). The magnified SEM images revealed small and large melanosomes beneath the C*β*P cortex; both contained air vesicles and were embedded in the C*β*P matrix (figure 1g).

We defined the structural parameters of Anna's hummingbird barbules as follows: (a) melanosome, (b) air vesicle, (c) melanosome membrane, (d) C*β*P, and (e) C*β*P cortex thickness (figure 1h). Melanosome layers of hummingbirds have typically been classified as either small melanosomes located directly below the cortex or larger melanosomes composing the underlying stack [31,40]. Our analysis identified a previously unreported intermediate size class of melanosomes (*N* = 199 melanosomes across 14 layers). The surface layer of the barbule consists of a thin C*β*P cortex and a layer of small, superficial melanosomes, collectively defined as the top layer. Beneath this region, two layers of intermediate-sized melanosomes form the middle layer, followed by multiple layers of larger melanosomes that constitute the bottom layer.

Figure 1i illustrates the average thickness of structural components within each melanosome layer, as determined from SEM measurements. These components include (a) total melanosome thickness, (b) internal air vesicle layer thickness, and (d and e) C*β*P layer thickness. The C*β*P layer represents the cortex overlying the melanosome array in the top melanosome layer, whereas in the middle and bottom layers, it refers to the interstitial regions between adjacent melanosomes. The measured melanosome layer thicknesses were 108 ± 5.4, 189 ± 15.6 and 222 ± 12.8 nm, respectively; detailed layer-by-layer analysis is presented in §3.4. The optical consequences of this three-layer architecture, and particularly the role of the intermediate melanosome layer in generating the secondary blue reflectance peak, are examined in the following sections.

### 3.2. Influence of the multi-periodic multi-layer on structural coloration

To test the hypothesis that the barbule nanostructure functions as a multi-periodic multi-layer, we performed optical simulations using wavelength-dependent complex refractive indices. Reflectance spectra were calculated with the FDTD method. Although this one-dimensional multi-layer structure could also be analysed using transfer-matrix methods, FDTD was adopted here to maintain methodological consistency with the subsequent three-dimensional tomography-based simulations performed within the same computational framework. The one-dimensional model-based simulation, consisting of alternating melanin, air and C*β*P layers, was constructed based on structural measurements from SEM images. The wavelength-dependent complex refractive indices of melanin and C*β*P were adopted from reported parameter values, while the effective refractive indices of the internal air vesicle layers were calculated based on the volume fractions of melanin, air and C*β*P. Details of parameters and calculation procedures are provided in the Methods section. We also reconstructed a three-dimensional structural model from serial SEM cross sections of the nanostructure (figure 2).

**Figure 2.**
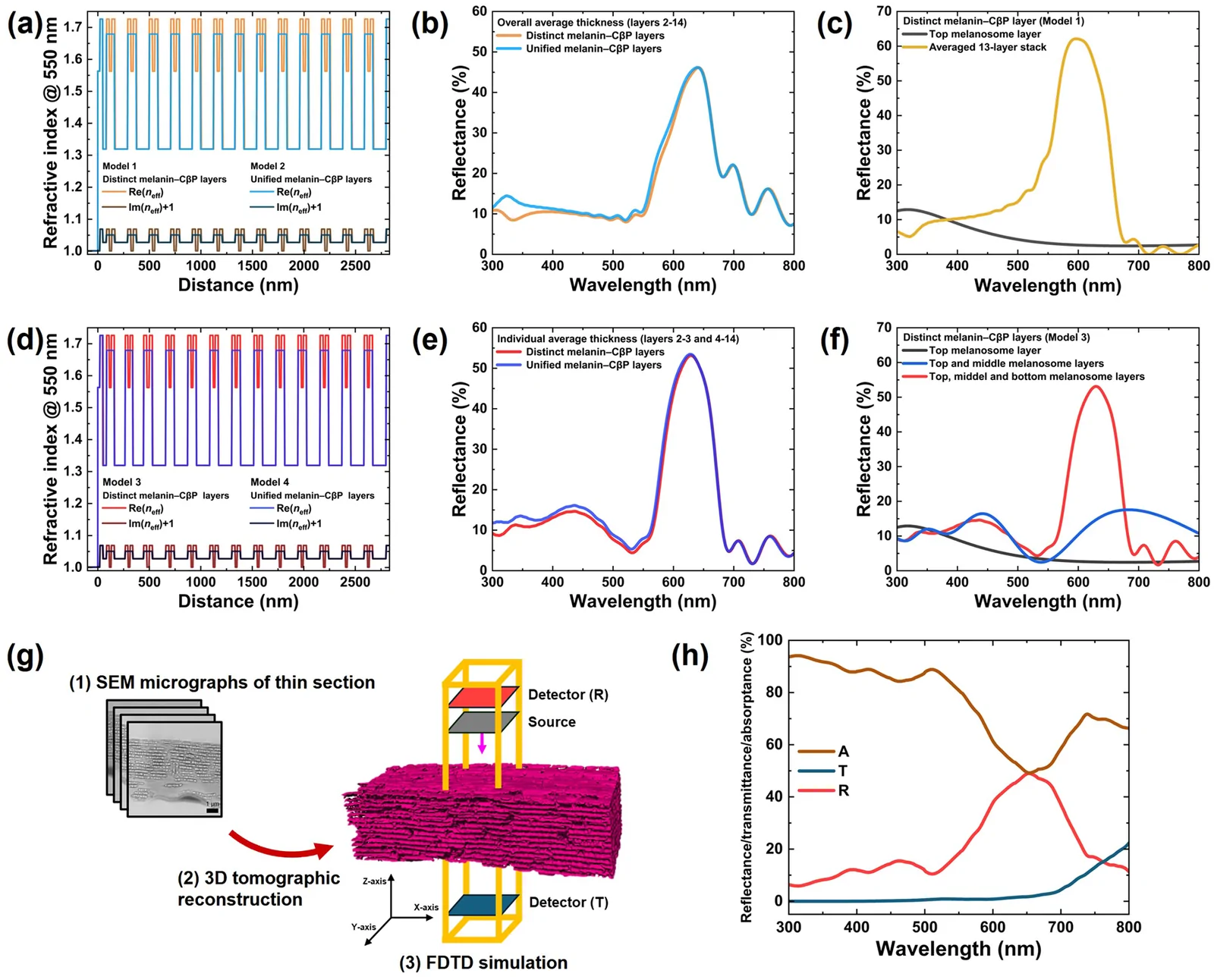
FDTD simulations of the multi-periodic multi-layer structure in Anna's hummingbird barbules. (a) Re (*n*_eff_) and Im (*n*_eff_) components of the effective refractive index (RI) at 550 nm for models 1 and 2, which use the overall average thickness of the middle and bottom melanosome layers. (b) Reflectance spectra of distinct melanin-C*β*P layers and unified melanin-C*β*P layers for models 1 and 2. (c) Reflectance spectra of the individual top and averaged melanosome layers in model 1, isolating their separate optical contributions. (d) Re (*n*_eff_) and Im (*n*_eff_) components of the effective RI at 550 nm for models 3 and 4, which use the individual averaged thickness of the middle and bottom melanosome layers. (e) Reflectance spectra of distinct melanin-C*β*P layers and unified melanin-C*β*P layers for models 3 and 4. (f) Cumulative layer analysis of model 3, demonstrating that the secondary blue peak emerges upon addition of the intermediate melanosome layers and persists in the full stack. (g) Three-dimensional tomographic reconstruction of the barbule nanostructure used in the FDTD simulation. (h) Absorptance (A), transmittance (T) and reflectance (R) spectra obtained from the three-dimensional FDTD simulation. Abbreviations: FDTD, finite-difference time-domain; C*β*P, corneous beta-protein; Re, real component; Im, imaginary component.

#### 3.2.1. One-dimensional model-based simulations

When optical simulations were conducted using the raw thickness values measured from the resin-embedded cross sections, the primary reflectance peak was simulated at 736 nm, substantially red-shifted relative to the measured value of 632 nm (supplementary material, figure S2). This discrepancy probably arises from geometric distortion during sample preparation, such as resin embedding and tilted cross-sectional slicing, which can overestimate layer thickness [30,31,41]. Consistent with multi-layer interference principles, such overestimation would shift the simulated peak toward longer wavelengths [42]. Therefore, we applied a scaling factor as an optical calibration to align the simulated primary peak with the microspectrophotometric measurement. Using a scaling factor of 85% yielded a simulated primary peak at 630 nm, in close agreement with the measured value of 632 nm. Because resin-free structural verification was not performed, this scaling should be regarded as a practical optical calibration rather than a directly validated geometric correction. To assess the robustness of our conclusions with respect to this scaling, simulations were performed across scaling factors from 80 to 100% (supplementary material, figure S2). As the scaling factor increased from 80 to 100%, the primary red peak shifted from 589 to 736 nm, and the secondary blue peak shifted from 409 to 510 nm. The multi-peak spectral profile and the distinct optical contribution of the intermediate melanosome layer were preserved across all tested values, confirming that the key findings reflect an intrinsic structural feature rather than an artefact of the scaling procedure.

Using the adjusted thickness values, FDTD simulations were conducted for four barbule nanostructure models, each consisting of 14 melanosome layers (table 1). In all models, the top layer contained a thin C*β*P cortex (21 nm) covering the small, superficial melanosome layer, with a membrane thickness of 29 nm and an intervening internal air vesicle layer of 34 nm.

**Table 1.** Layer thickness scaled to 85% and refractive indices (*n*, at 550 nm) used for each optical model and for the simplified optical thickness estimates.

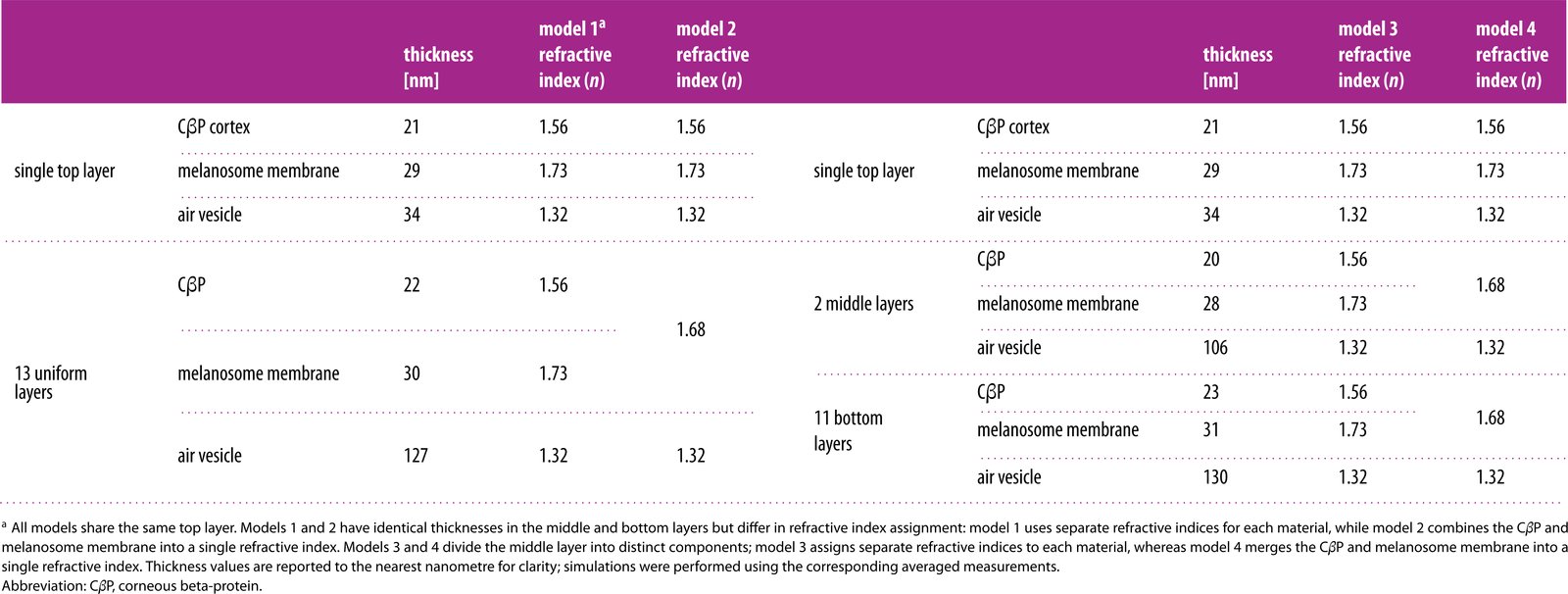

Models 1 and 2 were constructed with 13 melanosome layers of uniform thickness beneath the top layer (figure 2a). Each uniform layer was defined by the average dimensions of the middle and bottom layers. The structure consists of a 30 nm melanosome membrane, a 127 nm intervening air vesicle layer and a 22 nm C*β*P layer separating adjacent melanosome layers. The effective refractive index was calculated along the axis normal to the barbule surface (see Methods section for calculation details). The C*β*P cortex and inter-melanosomal C*β*P layers were treated as having a real refractive index, while the melanosome membrane and air vesicle layer containing melanin were modelled with complex refractive indices. In model 1, the melanosome membrane and C*β*P layer were treated as distinct components, each assigned a separate refractive index. By contrast, model 2 examined the effect of the high refractive-index contrast between melanin and the air vesicle layer on optical behaviour. For this model, optical simulations used effective refractive indices calculated from the relative thicknesses of melanosome membrane (73%) and C*β*P (27%), represented as alternating unified melanin-C*β*P and air vesicle layers beneath the top melanosome layer. Models 1 and 2 exhibited comparable reflectance spectra, with dominant red peaks at 640 and 642 nm, respectively (figure 2b). However, neither model reproduced a distinct secondary peak in the short-wavelength region. In model 1, the broadened blue sideband extended into the UV, whereas in model 2, the reflectance within this region increased further, deviating from the measured spectrum. These results indicate that models 1 and 2 fail to reproduce a discrete secondary blue peak. To clarify the optical roles of individual layers, we performed separate simulations for each layer. Figure 2c presents the simulated reflectance spectra of the top melanosome layer and the 13 averaged melanosome layers, including their associated C*β*P layers that constitute model 1. The single top melanosome layer beneath the C*β*P cortex only produced a broad reflectance band with a primary peak in the UV region (figure 2c). By contrast, the 13 uniform melanosome layers exhibited a primary peak at approximately 596 nm. When vertically stacked, these layers interact optically, causing slight shifts in the peak positions compared with those in the individual-layer simulations, while their respective optical contributions remain identifiable.

Models 3 and 4 were generated based on the structural classification using separately averaged thicknesses for the middle and bottom layers (figure 2d). The two middle layers comprised a 28 nm melanosome membrane, a 106 nm air vesicle layer and a 20 nm C*β*P layer between adjacent melanosomes, resulting in a periodicity of 182 nm. By contrast, the 11 bottom layers consisted of a 31 nm melanosome membrane, a 130 nm air vesicle layer and a 23 nm C*β*P layer between adjacent melanosomes, leading to a periodicity of 215 nm. Models 3 and 4 exhibited a red peak at 630 and 629 nm, respectively. Both models showed a minor blue peak at 436 nm, reproducing the multi-peak reflectance observed in optical measurements (figure 2e). These simulations underscore the optical significance of the middle layer, indicating that the intermediate-sized melanosomes play a crucial role in non-spectral colour production. The pronounced refractive index contrast between melanin and air is critical for generating the multi-peak reflectance pattern.

We further examined the cumulative build-up of the reflectance spectrum through cumulative layer simulations in model 3 (figure 2f). The top melanosome layer alone did not produce a distinct reflectance peak within the visible range. Upon addition of the two intermediate melanosome layers, two reflectance peaks emerged at 441 and 684 nm, indicating that the secondary blue peak arises from the optical interaction between the top and middle layers. The subsequent addition of the 11 bottom melanosome layers sharpened the red peak and shifted it from 684 to 630 nm, while the blue peak position remained stable at approximately 435 nm. Together, these results show that the multi-peak spectrum is a cumulative outcome of the hierarchical multi-layer structure, with the top and middle layers contributing to the shorter-wavelength feature and the bottom layers reinforcing the dominant red peak.

When the top layer of model 3 was modelled as a single homogeneous layer, approximated with a unified refractive index for the melanin-C*β*P constituents, as in models 2 and 4, a similar non-spectral reflectance was reproduced. Contour analysis of reflectance spectra as a function of top layer thickness revealed that multi-peak reflectance emerges only within a specific thickness range, identifying top layer thickness as a critical optical design parameter (supplementary material, figure S3). This thickness-dependent control is consistent with a cortex-like function, in which the top layer modulates the effective resonance conditions of the underlying periodic structure [43]. These simulations demonstrate that the multi-peak reflectance of Anna's hummingbird gorget feathers arises from the hierarchical interaction among all three-layer types: the secondary blue peak emerges from the optical interaction between the top and middle layers, while the bottom layers primarily reinforce the dominant red peak through multi-layer interference.

#### 3.2.2. Three-dimensional tomography-based simulations

A three-dimensional structural model was reconstructed from several SEM images of a cross-sectioned barbule nanostructure of Anna's hummingbird (figure 2g). The resulting three-dimensional image represents the internal air vesicle layer; however, limited contrast in the reconstruction prevented clear differentiation of the melanin and C*β*P regions. Simulations of models 2 and 4 demonstrated that combining the melanosome membrane with the C*β*P layer generates reflectance spectra similar to those obtained when the layers are treated separately. Therefore, the same refractive index values of the unified melanin–C*β*P layer from models 2 and 4 were applied to the three-dimensional nanostructure. Whereas the one-dimensional simulations were used to isolate the layer-specific optical contributions of the simplified multi-layer structure, the three-dimensional tomography-based simulations were performed to evaluate whether the same spectral behaviour is retained in a reconstructed nanostructure closer to the real barbule geometry. Although the one-dimensional system could also be analysed using transfer-matrix methods, FDTD was used for both to maintain methodological consistency across the two approaches. The FDTD method was used to calculate the reflectance and transmittance spectra of the three-dimensional nanostructure under normal incidence on the barbule (figure 2h). Absorptance was calculated as one minus the sum of reflectance and transmittance. This approach was used to reproduce the physical structure and to facilitate direct comparison of the simulated reflectance spectrum with the microspectrophotometric measurements (figure 1b). To isolate the optical role of the nanostructure, PMLs were incorporated, treating the substrate beneath the structure as a perfect absorber. The nanostructure was extended perpendicular to the direction of light propagation using periodic boundary conditions, while the PMLs absorbed and terminated the transmitted light. This lateral periodic continuation represents an idealized approximation of the reconstructed local nanostructure and may introduce artificial coherence in the in-plane direction; however, because the dominant spectral response is governed primarily by the stacking-direction multi-layer architecture, we do not expect this approximation to qualitatively alter the main optical conclusions.

One-dimensional model-based FDTD simulations revealed how the multi-periodic multi-layer structure generates the coloration of Anna's hummingbird, and subsequent three-dimensional FDTD simulations based on tomographic structural models validated these findings. The three-dimensional simulation reproduced the same reflectance trend observed in the feather, with a dominant peak at 653 nm in the red region, a secondary peak at 462 nm in the blue region and a weak rise less than 400 nm extending into the ultraviolet. The transmittance spectrum shows a pronounced increase with wavelength attributable to the reduced absorption of melanin at longer wavelengths [37]. Absorptance gradually decreases from the short- to the long-wavelength region, reaching its minimum near the red reflectance peak. This trend reflects the combined effects of melanin absorption and nanostructural reflectance. Beyond the primary reflectance peak, the relatively high absorptance at shorter wavelengths potentially contributes to the enhanced saturation of the magenta coloration observed in Anna's hummingbird [2].

These optical simulations have some limitations. First, in the simulation with one-dimensional optical modelling, the internal vesicles within each melanosome were represented by a layer with a calculated refractive index, rather than discrete spheres. As a result, the specific optical effects caused by the spatial distribution of the air vesicles could not be fully analysed. Nevertheless, the overall agreement between the simulated and measured reflectance spectra suggests that these vesicles primarily act as low-refractive-index components contributing to the refractive contrast, rather than significantly altering the spectral shape. Second, in the three-dimensional tomographic reconstruction, the segmentation process was performed using a single contrast threshold to distinguish solid and void regions. Consequently, melanin and C*β*P were not differentiated and were treated as a single material. Although the comparison among one-dimensional models indicated that this approximation introduces minimal differences in the main reflectance peaks, minor deviations arising from the refractive index contrast between melanin and C*β*P cannot be completely excluded. Nevertheless, distinct red and blue reflectance peaks were successfully reproduced, confirming that the layer thickness of each constituent plays a dominant role in determining the reflectance characteristics.

### 3.3. Optical function of the top layer in structural coloration

In our previous simulations, the top layer alone did not produce a distinct reflectance peak within the visible range (figure 2c,f). However, its position at the outermost surface facilitates optical interactions with the underlying nanostructures, thereby influencing the overall coloration [39,44,45]. To investigate the optical effect of the top layer, we performed oxygen plasma etching to remove these top layers, adjusting the thickness reduction by varying the plasma exposure duration (see Methods section for details), and subsequently analysing the resulting structural changes using FDTD, as shown in figure 3.

**Figure 3.**
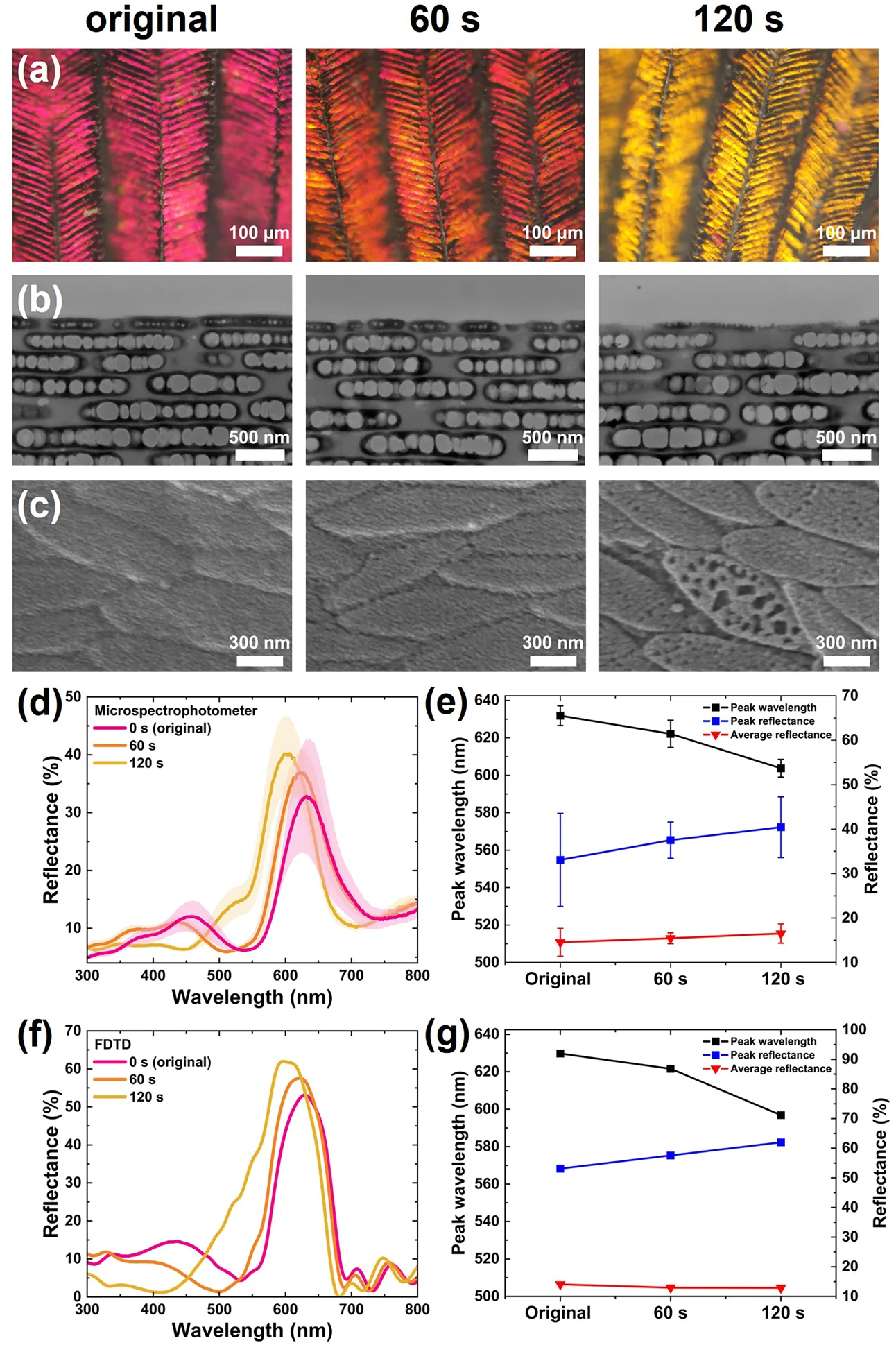
Optical and structural changes following top-layer etching. (a) Optical images of unetched and etched barbules after 60 and 120 s of etching. (b) Cross-sectional SEM images of barbules at different etching durations. (c) Top-view SEM images illustrating surface morphology changes with etching duration. (d) Measured reflectance spectra of barbules obtained using a microspectrophotometer. (e) Peak wavelength and reflectance values of barbules (*N* = 12, 11 and 14 for 0, 60 and 120 s etching, respectively); error bars indicate s.d. (f) Simulated reflectance spectra of the barbule nanostructure (model 3) obtained using FDTD simulations incorporating top-layer etching-induced structural modifications. (g) Simulated peak wavelength and reflectance values obtained from FDTD simulations. Abbreviations: SEM, scanning electron microscopy; FDTD, finite-difference time-domain.

As the plasma etching duration increased from 0 to 60 and 120 s, the barbule coloration gradually shifted from magenta to reddish-orange and finally to yellowish-orange (figure 3a). The corresponding cross-sectional SEM images (figure 3b) clearly revealed the near-complete removal of the C*β*P cortex after 60 s of etching and additional removal of the melanosome membrane after 120 s. Additionally, the corresponding top-view SEM images revealed these structural changes. After 60 s of etching, the barbule exhibited more pronounced interspaces between adjacent melanosomes than the original unetched sample, while the 120 s etched barbules further revealed air vesicles formed after the removal of the melanosome membrane (figure 3c). Microspectrophotometry revealed that, relative to the original magenta barbule, the primary red reflectance peak progressively shifted towards shorter wavelengths, moving from 632 to 622 and 604 nm as the etching duration increased (figure 3d). This progressive blue shift (i.e. hypsochromic shift) is consistent with a reduction in the effective optical thickness of the multi-layer and with changes in the reflection phase at the superficial interface following removal of the top layer, both of which would shift the dominant long-wavelength reflectance toward shorter wavelengths [39,45,46]. Concurrently, the peak reflectance increased from 32.8 to 37.5 and 40.4% for the barbules etched for 0, 60 and 120 s, respectively (figure 3e). The corresponding average reflectance values were 14.5, 15.4 and 16.5% for the barbules etched for 0, 60 and 120 s, respectively. Additionally, the secondary blue reflectance peak shifted from 459 to 437 nm, while its reflectance decreased and eventually vanished after 120 s of etching. The extinction of the secondary blue peak upon complete removal of the top melanosome layer is consistent with the optical interaction mechanism established in figure 2f: the blue peak originates from the optical interaction between the top and middle layers, and its disappearance indicates that the top layer is required for blue peak generation. This behaviour cannot be explained by loss of melanin absorption alone, because removal of a broadband absorber would be expected to increase, rather than suppress, short-wavelength reflectance.

To quantitatively evaluate the optical effects of structural variations in the top layer, we conducted FDTD-based simulations. Consistent with the experimental observations, changes in the top layer influenced the coloration of the feather barbules. The simulations were performed using optical model 3, incorporating distinct melanin-C*β*P layers and averaging thickness values to represent the structural changes observed after 60 s of etching (cortex removal) and after 120 s (elimination of the top melanosome membrane layer). Consistent with the experimental results, the primary red peak exhibited a blue shift from 629 to 621 and 596 nm as the layer was progressively removed, while its reflectance increased from 53.1 to 57.6 and 62.0% (figure 3f,g). By contrast, the corresponding average reflectance values decreased to 14.0, 13.0 and 12.9% for the modelled barbules etched for 0, 60 and 120 s, respectively. Furthermore, the secondary blue peak decreased in reflectance and eventually vanished after 120 s of etching. These FDTD simulation results support the interpretation that experimentally observed spectral shifts arise from structural modifications of the top layer, which influence the effective optical path length of the multi-layer stack. However, the simulated average reflectance showed a slight decrease with progressive etching time, whereas the measured values increased; this discrepancy may reflect simplifications inherent to the idealized planar model, including the neglect of surface roughness and non-uniform etching.

However, these changes are owing to the top layer's properties and its interaction with the layers below. To investigate this relationship, we performed simulations for two configurations: one containing only the top layer and another comprising both the top and middle layers, and then evaluated structural variations in the top layer to determine their effect on reflectance (supplementary material, figure S4). When only the top layer was present, structural modifications led to an overall decrease in reflectance intensity across the 300–400 nm wavelength range. However, when both the top and middle layers were included, the unmodified structure exhibited two distinct reflectance peaks in the red and blue regions. Sequential removal of the C*β*P cortex (60 s) and the top melanosome membrane (120 s) caused a blue shift of the red peak along with an increase in its reflectance intensity. By contrast, the blue peak also shifted toward shorter wavelengths but exhibited reduced intensity, while reflectance in the near-UV region similarly decreased. When both the C*β*P cortex and top melanosome membrane were removed, only a single dominant peak remained at 567 nm. The appearance of this single peak is consistent with the increased reflectance observed in the 450–550 nm range after 120 s of etching (figure 3d,f). These findings indicate that the top layer influences the overall colour saturation and brightness, while the optical interaction with the adjacent middle layer produces the secondary peak.

### 3.4. Secondary blue peak as an optical consequence of the graded nanostructure in barbules

Structural measurements along the stacking direction (Z-axis) revealed a clear size gradient from the barbule surface to the interior (figure 4a,e; supplementary material, table S1). A previous study reported a similar trend in melanosome length along the Y-axis [36]. The outermost melanosome (layer 1) had a mean thickness of approximately 110 nm, substantially thinner than layers 2 and 3 (approx. 180–200 nm) and the deeper layers 4–14 (approx. 220–240 nm), where thickness stabilized. This layer-dependent variation was driven primarily by air vesicle dimensions (figure 4b): the air vesicle of the top melanosome (approx. 40 nm) was markedly smaller than those of the middle (approx. 120 nm) and bottom layers (approx. 140–160 nm), whereas melanosome membrane thickness remained comparatively uniform across layers (figure 4c). The C*β*P layer thickness between adjacent melanosomes was also relatively uniform (approx. 20–26 nm), except for the interface between melanosomes 4 and 5, which was distinctly thicker (approx. 42 nm) (figure 4d). These raw structural measurements support the three-layer optical classification adopted in this study: top (layer 1), middle (layers 2 and 3) and bottom (layers 4–14).

**Figure 4.**
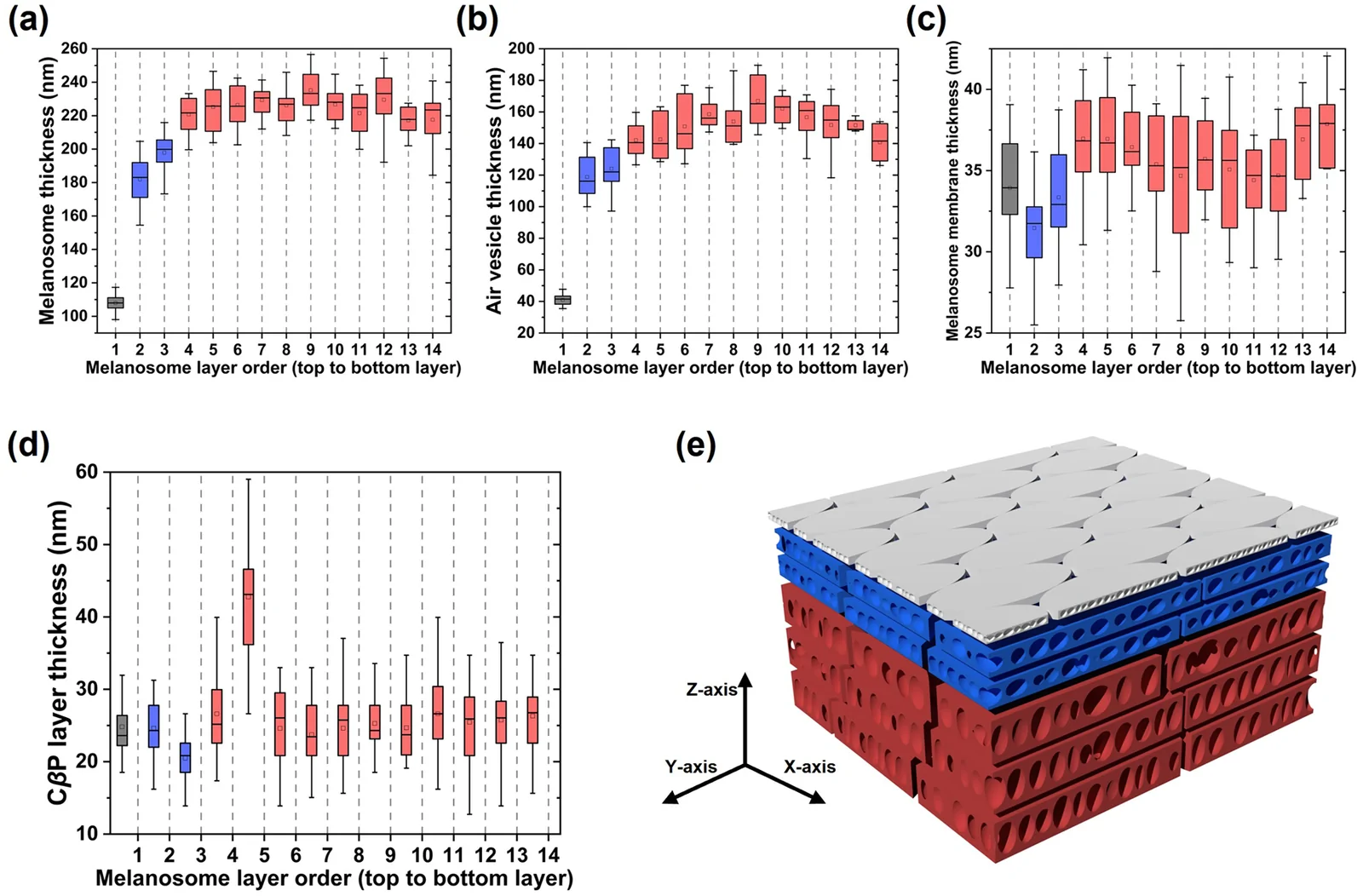
Variation in nanostructural component thicknesses across the barbule layers. (a) Melanosome thickness across successive layer positions. (b) Air vesicle thickness as a function of layer position. (c) Melanosome membrane thickness as a function of layer position. (d) C*β*P layer thickness measured above and between the melanosome layers. (e) Schematic illustration of the different melanosome layer types. Abbreviation: C*β*P, corneous beta-protein.

Optical-thickness estimates based on the scaled structural parameters provide qualitative support for this interpretation, suggesting that the combined periodicity of the top and middle layers gives rise to a short-wavelength resonance that neither layer alone can produce (see supplementary material for details). Consistent with this interpretation, the superficial top region alone does not produce a distinct reflectance maximum in the visible range, whereas the blue peak near 441 nm is reproduced only when the top and middle layers are considered together in the FDTD simulations (figure 2f). These results indicate that the blue reflectance of Anna's hummingbird gorget feathers cannot be explained by the top or middle layer alone but instead arises from optical interaction between the superficial top layer and the intermediate melanosome layers within the graded melanosome architecture identified in this study.

The developmental origin of this size gradient remains uncertain, but it may reflect physical constraints during progressive melanosome stacking and C*β*P cornification [36]. Melanosomes that migrate to and align along the barbule edges are thought to undergo self-assembly driven by depletion attraction [47], a process described by the Asakura–Oosawa model, in which overlapping excluded volumes generate an unbalanced osmotic pressure that effectively draws the larger particles together [48–50]. Progressive hardening of the surrounding matrix may further increase crowding pressures on superficial melanosomes, thereby limiting vesicle expansion. Regardless of its developmental origin, the observed size gradient defines three optically distinct melanosome classes whose hierarchical arrangement enables the secondary blue reflectance peak and, together with the deeper multi-layer stack, gives rise to the non-spectral magenta coloration. Thus, the secondary blue peak in Anna's hummingbird gorget can be understood as an emergent optical consequence of the graded nanostructure.

## 4. Conclusion

In summary, electron microscopy and tomographic analyses revealed a continuous size gradient of melanosomes across the barbule depth in Anna's hummingbird, with a single small superficial melanosome and progressively larger melanosomes in deeper layers. Based on their distinct periodicities, these melanosomes were divided into three layers. Optical simulations based on one-dimensional and three-dimensional modelling demonstrated that this multi-periodic arrangement functions as a hierarchical multi-layer reflector, in which the secondary blue peak is generated by the optical interaction between the top and middle layers, while the bottom layers primarily produce the dominant red peak through multi-layer interference. Neither layer alone was sufficient; blue peak generation required their combined optical interaction. Oxygen plasma etching further supported this mechanism by showing that removal of the top layer eliminated the secondary blue peak. Structural measurements further revealed that the three-layer classification reflects a continuous size gradient in melanosome dimensions, which probably arises from physical constraints during barbule development. Together, these findings establish that the non-spectral magenta coloration of Anna's hummingbird gorget results from the hierarchical interaction of three structurally distinct melanosome classes. Although the present study establishes the mechanism underlying non-spectral coloration in Anna's hummingbird arising from its multi-periodic multi-layer architecture, it remains an open question whether similar structures underlie non-spectral coloration in other hummingbird species. In addition, the developmental origin of the continuous melanosome size gradient inferred here remains to be directly resolved. This multi-periodic design principle may inform the rational design of biomimetic nanophotonic structures capable of generating tunable non-spectral colours for applications in displays, sensors and anti-counterfeiting materials.

## Supplementary Material



## Data Availability

All data supporting this article are provided as electronic supplementary material, which is available online. Supplementary material is available online [51].
