# Supplementary material for: Multi-periodic photonic mechanism for non-spectral magenta coloration in Anna's hummingbird (Calypte anna) feathers
Source: J R Soc Interface. 2026 Oct 28;23(243):20260601. doi: 10.1098/rsif.2026.0601 (PMC13714409; doi:10.1098/rsif.2026.0601)
Supplement: Supplementary file 1 [file rsif.2026.0601.zip › rsif.2026.0601/rsif20260601_si_001.docx]

**Supporting Information**

Multiperiodic Photonic Mechanism for Nonspectral Magenta Colouration in Anna's Hummingbird (*Calypte anna*) Feathers

Sangyeop Kim^1 ,2^, Jihun Kang^1 ,2^, Seunghwan Moon^1 ,2^, Liliana D'Alba^3, 4^, Matthew D. Shawkey^3^, and Jong-Souk Yeo^1 ,2^*

E-mail: jongsoukyeo@yonsei.ac.kr

Article DOI: 10.1098/rsif.2026.0601

^1^School of Integrated Technology, College of Computing, Yonsei University, Incheon 21983, Republic of Korea

^2^BK21 Graduate Program in Intelligent Semiconductor Technology, Yonsei University, Seoul 03722, Republic of Korea

^3^Evolution and Optics of Nanostructures Group, Department of Biology, Ghent University, Ledeganckstraat 35, Ghent 9000, Belgium

^4^Naturalis Biodiversity Center, Darwinweg 2, Leiden 2333 CR, The Netherlands

**Contents of this Supporting Information**

- Figures S1-S4
- Table S1
- Supplementary Note

**Figure S1**. Structural characterisation of barbule nanostructures used for thickness measurements. Melanosome thickness (a) was determined based on the average of multiple measurements across different positions within a single organelle. Air vesicle thickness (b) was measured similarly from vesicles observed within the same melanosome cross-section. The melanosome membrane thickness (c) was calculated as (a − b)/2. CβP layer thickness (d) was calculated based on averaging multiple measurements between adjacent melanosomes, and cortex thickness (e) was measured from the outer surface to the underlying melanosomes.

**Abbreviation**: CβP*,* corneous beta-protein

**Figure S2**. a) FDTD-simulated reflectance spectra of Anna’s hummingbird barbule nanostructures with thicknesses varied from 80% to 100% and b) simulated normalised reflectance spectra compared with the measured result. The 1D model-based optical simulation accounted for the melanosome membrane, air vesicle layer, and CβP layer thicknesses, starting from the outermost CβP cortex.

**Abbreviations**: FDTD, finite-difference time-domain; CβP*,* corneous beta-protein

**Figure S3**. Simulation parameters and results of the modified Model 3 with a single homogeneous top layer. (a) Re (n_eff_) and Im (n_eff_) components of the effective RI at 550 nm for the Model 3 variant. The complex refractive index of the single homogeneous top layer (113 nm) was calculated by considering the thickness-weighted proportions of each constituent material. (b) Corresponding reflectance spectrum of the Model 3 variant. (c) Colour contour plots of the reflectance spectra as a function of the homogeneous top layer thickness for the modified Model 3 (blue: minimum, red: maximum), indicating that multi-peak reflectance in the red and blue regions occurs only when the top layer has an appropriate thickness.

**Figure S4**. Optical simulation results showing the effects of top-layer structural modifications via etching. a) Reflectance spectra of the top layer: with CβP cortex and a single small melanosome (0 s), without CβP cortex (60 s), and without CβP cortex and the top melanosome membrane (120 s). b) Reflectance spectra of the stacked structure of the top and middle layers: with CβP cortex (0 s), without CβP cortex (60 s), and without CβP cortex and the top melanosome membrane (120 s).
**Abbreviation**: CβP*,* corneous beta-protein

**Table S1**. Quantitative structural analysis of the barbules in Anna’s hummingbird gorget feathers.

| Barbule structural components | 1 | 2 | 3 | 4 | 5 | 6 | 7 | 8 | 9 | 10 | 11 | 12 | 13 | 14 |
| --- | --- | --- | --- | --- | --- | --- | --- | --- | --- | --- | --- | --- | --- | --- |
| Melanosome [nm] | 108  (5.4) | 182  (14.2) | 198  (12.8) | 221  (10.9) | 225  (14.3) | 226  (12.9) | 229  (8.3) | 226  (10.5) | 235  (11.9) | 227  (11.1) | 222  (12.0) | 230  (16.9) | 217  (8.4) | 218  (16.1) |
| Air vesicle [nm] | 40  (3.5) | 119  (14.3) | 131  (12.0) | 147  (11.7) | 151  (15.2) | 154  (15.8) | 159  (11.5) | 157  (16.0) | 164  (13.4) | 157  (11.1) | 152  (13.7) | 160  (20.4) | 143  (9.0) | 142  (16.8) |
| Melanosome membrane [nm] | 34  (3.0) | 32  (3.1) | 33  (3.1) | 37  (2.8) | 37  (3.0) | 37  (2.3) | 35  (2.7) | 35  (4.6) | 36  (2.6) | 35  (3.5) | 34  (2.3) | 35  (2.7) | 37  (2.5) | 38  (2.4) |
| CβP cortex,  CβP [nm] | 25  (3.8) | 25  (4.0) | 21  (3.3) | 27  (6.3) | 43  (8.1) | 25  (4.9) | 24  (5.2) | 25  (5.0) | 25  (3.7) | 25  (4.5) | 27  (5.7) | 25  (5.8) | 26  (5.1) | 26  (4.4) |

Nanostructural components within the barbules were measured from high-magnification (×40,000 and ×100,000) cross-sectional SEM images using ImageJ software. Each value represents the average, with the numbers in parentheses indicating the standard deviation. Melanosome and air vesicle thickness were measured from *N* = 199, averaging 14 measurements per layer. Melanosome membrane thickness was calculated as (melanosome thickness – air vesicle thickness)/2. CβP cortex and CβP were measured from *N* = 403, averaging 29 measurements per layer.

**Abbreviation:** CβP*,* corneous beta-protein; SEM, scanning electron microscopy

**Supplementary Note**: Optical-thickness estimates for the top and middle melanosome layers

Using the scaled thicknesses and refractive indices summarised in Table 1 of the main text, the characteristic optical thickness of the multilayer can be approximated using the constructive interference relation for a multilayer stack,

$m\lambda=2\sum_{i} n_{i}d_{i}$,

when $n_{i}$ and $d_{i}$ are the refractive index and thickness of each sublayer [1]. For one intermediate period (Table 1),

$$\sum_{i} n_{i}d_{i}=(1.56\times20)+(1.73\times28)+(1.32\times106)+(1.73\times28)\approx268 nm$$

Assuming first-order constructive interference (m = 1), this corresponds to

$\lambda\approx2\sum_{i} n_{i}d_{i}\approx536 nm$,

which does not match the simulated secondary blue peak near 441 nm. This indicates that the intermediate layers alone cannot account for the short-wavelength feature. Using the corresponding scaled parameters for the superficial top layer listed in Table 1 gives

$$\sum_{i} n_{i}d_{i}=(1.56\times21)+(1.73\times29)+(1.32\times34)+(1.73\times29)\approx178 nm$$

The combined top and middle stack has a total optical thickness of approximately 446 nm. Notably, the second-order mode (m = 2) of this combined stack predicts a resonance near 446 nm, consistent with the blue peak at 441 nm observed in the FDTD simulation. While this agreement is suggestive, the resonance order is not independently constrained by the present data; this calculation therefore serves as an intuitive optical-thickness argument rather than a strict mechanistic assignment, illustrating why the middle layer alone cannot account for the blue peak and why inclusion of the top layer is required to generate a short-wavelength response. For such arbitrary multilayer stacks, the optical response is more generally calculated using transfer-matrix or full-wave methods [2-6].

**References**

[1] Matin, T.R., Leong, M., Majlis, B.Y. & Gebeshuber, I.C. 2010 Correlating nanostructures with function: Structural colors on the wings of a Malaysian bee. In *AIP Conference Proceedings* (pp. 5-14, American Institute of Physics.

[2] Vignolini, S., Moyroud, E., Glover, B.J. & Steiner, U. 2013 Analysing photonic structures in plants. *Journal of the Royal Society Interface* **10**. (doi:10.1098/rsif.2013.0394).

[3] Schertel, L., van de Kerkhof, G.T., Jacucci, G., Catón, L., Ogawa, Y., Wilts, B.D., Ingham, C.J., Vignolini, S. & Johansen, V.E. 2020 Complex photonic response reveals three-dimensional self-organization of structural coloured bacterial colonies. *Journal of the Royal Society Interface* **17**. (doi:10.1098/rsif.2020.0196).

[4] Katsidis, C.C. & Siapkas, D.I. 2002 General transfer-matrix method for optical multilayer systems with coherent, partially coherent, and incoherent interference. *Applied optics* **41**, 3978-3987. (doi:10.1364/AO.41.003978).

[5] Sreekanth, K.V., Zeng, S., Shang, J., Yong, K.-T. & Yu, T. 2012 Excitation of surface electromagnetic waves in a graphene-based Bragg grating. *Scientific reports* **2**, 737. (doi:10.1038/srep00737).

[6] McCoy, D.E., Shneidman, A.V., Davis, A.L. & Aizenberg, J. 2021 Finite-difference time-domain (FDTD) optical simulations: a primer for the life sciences and bio-inspired engineering. *Micron* **151**, 103160. (doi:10.1016/j.micron.2021.103160).
